# Hepatoprotective effect of feeding celery leaves mixed with chicory leaves and barley grains to hypercholesterolemic rats

**DOI:** 10.4103/0973-1296.80675

**Published:** 2011

**Authors:** Nehal M. Abd El-Mageed

**Affiliations:** *Department of Nutrition and Food Science, Faculty of Home Economics, Helwan University, Egypt*

**Keywords:** Barley, biochemical analysis, celery, chicory, hypercholesterolemia

## Abstract

Celery, chicory leaves, and barley grains are valuable in weight loss diets and regulate lipid metabolism. They may reduce risk of fatty liver. The present study aimed to investigate the effect of diet supplementation with celery, chicory, and barley powder on liver enzymes and blood lipids in rats fed with cholesterol-enriched diet. This study used four groups of rats fed with 3% cholesterol were supplemented diet to induce hypercholesterolemia and one group was fed on cholesterol-free basal diet. The dry powder of celery leaves, chicory leaves, and barley grains was separately added to the basal diet at 10% concentration or in combination of three plants at 15% for four weeks. Biochemical analyses of serum liver enzymes and blood lipids as well as histopathological examination of liver were performed. Feeding of diet supplemented with 10% of celery, 10% chicory, and 10% of barley lowered the elevated serum level of liver enzymes and blood lipids in rats. Feeding plant combination of celery, chicory, and barley at 15% concentration (5% from each) was more effective in decreasing the elevation of liver enzymes (aspartate aminotransferase, alanine aminotransferase, and alkaline phosphatase) and blood lipids. The histopathological lesions seen in the livers of hypercholesterolemic rats were ameliorated by feeding this plant mixture. This study recommends that dietary intake of plant mixture of celery; chicory, and barley at 15% (5% of each) concentration can be beneficial to patients suffering from hypercholesterolemia and liver diseases.

## INTRODUCTION

Celery (*Apium graveolens*, Family *Apiaceae*) is an excellent source of vitamin C. It is a very good source of dietary fiber, potassium, folate, manganese, and vitamin B6. Celery is also a good source of calcium, vitamin B1, vitamin B2, magnesium, vitamin A, phosphorus, and iron.[1]

Chicory (*Cichorium intybus*, Family *Asteraceace*) chicory extracts and/or formulations containing the roots or leaves revealed that they produce hepatoprotective[2,3] and antioxidant effects.[4,5]

Inulin (fructans) extracted from chicory regulates appetite and lipid/glucose metabolism. It has also promising effects on the body weight and fat mass development.[6]

Barley (*Hordeum vulgare* L, Family *Poaceae*) makes a natural choice for healthful benefits as it is rich in protein, carbohydrates, dietary fibers, chromium, fat, and it is cholesterol free.[7] Moreover, barley may be used as a part of vegetarian diet, because it decreases total lipids and reduces the risk of developing liver disease, such as fatty liver.[8]

The present study aimed to investigate the effect of diet supplementation with celery, chicory, and barley powder on liver enzymes and blood lipids profile in hypercholesterolemic rats.

## MATERIALS AND METHODS

### Materials

#### Plants

Celery and Chicory leaves were from obtained a local market of Herbs and Medicinal plants, Cairo, Egypt. Barley grains (Giza 128 variety) were obtained from Agricultural Research Center, Giza, Egypt. The selected plant materials were air-dried, grinded in an electrical blender into a fine powder which was packed in air-tight plastic bags till use for basal diet supplementation. The chemical composition of plants used is shown in Table 1.

**Table 1:** Shows the chemical composition per 100 g of edible plants

Amount in plants			Nutrient
Chicory	Barley	Celery	
15 kcal	350 kcal	12 kcal	Energy
4 g	73.7 g	3.6 g	Carbohydrate
0.0 g	9.9 g	0.84	Protein
1.0 g	14.9 g	2.04 g	Dietary fiber
0.0 g	1.2 g	0.24	Fats
200.0 mg	0.0 mg	8.40 mg	Vitamin C
Traces	23 μg	33.60 μg	Folate
Traces	0.3 mg	0.10 mg	Vitamin B6 (pyridoxine)
Traces	0.2 mg	0.06 mg	Vitamin B1 (thiamin)
Traces	0.1 mg	0.05 mg	Vitamin B2 (riboflavin)
Traces	Traces	160.80 IU	Vitamin A
Traces	Traces	35.26 μg	Vitamin K
136 mg	280 mg	344.4 mg	Potassium
Traces	Traces	0.12 mg	Manganese
200.0 mg	29.0 mg	48.00 mg	Calcium
Traces	221 mg	30.00 mg	Phosphorus
Traces	79.0 mg	13.20 mg	Magnesium
Traces	2.5 mg	0.48 mg	Iron

Determination of the nutritional value in the Agricultural Research Center, Giza, Egypt, Percent daily values are based on a 2 000 calorie diet

#### Cholesterol

It was purchased from El-Gomhuryia Company for Chemical Industries, Cairo.

#### Rats

Adult male albino rats of Sprague Dawley strain weighing 150 to 160 g body weight were used in this study.

### Methods

#### Preparation of basal diet

Basal diet was prepared. It consists of 20% protein (casein), 10% sucrose, 4.7% corn oil, 2% choline chloride, 1% vitamin mixture, 3.5% salt mixture, and 5% fibers (cellulose). The remainder was corn starch.[9]

#### Induction of hypercholesterolemia

It was induced by feeding rats on basal diet supplemented with 3% cholesterol for four weeks before start of the experiment. After feeding period, a random blood sample was withdrawn from the orbital sinus of the eye and serum total cholesterol was measured to insure that hypercholesterolemia was induced.[10]

#### Experiments and grouping of rats

Forty-two male albino rats were used in this experiment in laboratory animals, depending on the animal ethical Committee. Rats were divided into 6 equal groups of 7 animals each. Group (1) was fed on the basal diet as a negative control group, while the other groups were fed on 2% cholesterol-supplemented diet for four weeks for induction of hypercholesterolemia. Group (2) was left as a positive control (hypercholesterolemic), while groups (3), (4), (5), and (6) were fed on experimental diets containing 10% celery, 10% chicory, 10% barley, and 15% mixture (5% from each) of celery (5% celery = 22.7 g edible weight), chicory (5% chicory = 18.5 g edible weight), and barley, respectively, for four weeks. During the feeding period, body weight gains (BWG%) and food efficiency ratios (FERs) were calculated.[11]

At the end of experimental period, the rats were anesthetized by ether and blood samples were collected. Blood samples were centrifuged for 20 minutes at 3 000 rpm to separate the serum which was kept at -10°C till biochemical analysis of liver enzymes, total cholesterol, triglycerides, and lipoprotein fractions. Livers of the sacrificed rats were removed and preserved in 10% neutral formalin solution till histopathological examination.

#### Biochemical analyses

The collected serum samples were used for estimation of aspartate and alanine aminotransferases (AST and ALT) enzymes[12] and alkaline phosphatase (ALP).[13]

Serum total cholesterol and triglycerides were calorimetrically determined.[14,15]

High-density lipoprotein cholesterol (HDL-c) was calorimetrically determined, very low-density lipoprotein cholesterol (VLDL-c) and low-density lipoprotein cholesterol (LDL-c) were mathematically calculated.[16]

#### Histopathological examination

Livers of the scarified rats were dissected, removed, and fixed in 10% formalin solution. The fixed specimens were then trimmed, washed, and dehydrated in ascending grades of alcohol. These specimens were cleared in xylene, embedded in paraffin, sectioned at 4-6 μ of thickness and stained with Hematoxylin and Eosin, then examined microscopically.[17]

### Statistical analysis

Results are expressed as mean values with their standard deviation of the mean. Statistical differences between groups were evaluated using one-way ANOVA followed by Duncan post hoc test using SPSS version 11.0 for Windows (SPSS, Chicago, IL, USA).[18]

## RESULTS

As shown in Table 2, feeding normal rats on basal diet supplemented with cholesterol for four weeks significantly increased BWG% and FER. Feeding hypercholesterolemic rats on diet supplemented with 10% celery significantly decreased BWG% and FER, whereas diets supplemented with 10% chicory or 10% barley or 15% mixture of the three plants caused significant increases in BWG% and FER.

**Table 2:** Effect of diet supplementation with celery, chicory or barley and their combination on food intake, body weight gain and food efficiency ratio in hypercholesterolemic rats. (n = 7 rats)

Groups	FI (g)	BWG (%)	FER
Negative Control	9.3 ± 0.40^a^	10.2 ± 0.25^a^	1.09a
Positive Control	13.2 ± 0.50^c^	16.9 ± 0.32^c^	1.28bc
Celery (10%)	11.2 ± 0.10^b^	12.2 ± 0.13^b^	1.08a
Chicory (10%)	13.9 ± 0.80^cd^	17.0 ± 0.13^c^	1.22b
Barley (10%)	14.4 ± 0.20^d^	18.1 ± 0.41^d^	1.25b
Mixture of all (15%)	14.5 ± 0.10^d^	18.9 ± 0.32^d^	1.30c

Values are mean ± SD. Values in the same column sharing the same superscript letters are not statistically significantly different, FI: Food intake, BWG: Body weight gain, FER: Food efficiency ratio

It is clear from Table 3 that feeding of diet supplemented with celery, chicory, and barley plant powder, alone and combined, for four weeks to hypercholesterolemic rats significantly decreased the levels of AST, ALT, and ALP enzymes in the serum, compared with the control positive group.

**Table 3:** Effect of diet supplementation with celery, chicory or barley and their combination on serum aspartate aminotransferase, alanine aminotransferase and alkaline phosphatase enzymes in hypercholesterolemic rats. (n = 7 rats)

Groups	AST (U/l)	ALT (U/l)	ALP (U/l)
Negative control	63.5 ± 2.6^a^	26.5 ± 1.6^a^	90.98 ± 1.4^a^
Positive control	75.4 ± 3.2^d^	37.5 ± 2.2^c^	104.95 ± 1.6^d^
Celery (10%)	72.6 ± 2.4^bc^	34.5 ± 3.3^b^	100.97 ± 1.8^c^
Chicory (10%)	71.3 ± 2.3^b^	33.7 ± 3.6^b^	98.90 ± 1.2b^c^
Barley (10%)	66.9 ± 3.8^ab^	32.1 ± 3.9^b^	96.16 ± 1.3^b^
Mixture of all (15%)	64.5 ± 2.8^a^	28.1 ± 3.9^ab^	92.00 ± 1.5^a^

Values are mean ± SD. Values in the same column sharing the same superscript letters are not statistically significantly different, AST: aspartate aminotransferase, ALT: Alanine aminotransferase, ALP: Alkaline phosphatase

As demonstrated in Table 4, feeding experimental diets supplemented with celery, chicory, and barley plant powder, alone and combined, for four weeks to hypercholesterolemic rats significantly decreased levels of total cholesterol and triglycerides in the serum, as compared with the control positive group. Feeding hypercholesterolemic rats on diets supplemented with celery, chicory, and barley plant powder caused a significant decrease in serum level of LDLc and VLDLc, but significantly increased the levels of HDLc. As it is well known that LDLc is bad cholesterol, these plant material when added to basal diet improved lipid profile.

**Table 4:** Effect of diet supplementation with celery, chicory or barley and their combination on serum lipoprotein fractions, total cholesterol and triglyceride in hypercholesterolemic rats. (n = 7 rats)

Groups	Lipoproteins (mg/dl)			Total Cholesterol (mg/dL)	Triglycerides (mg/dl)
	HDL-c	LDL-c	VLDL-c
Negative control	46.60 ± 0.19^c^	38.20 ± 0.21^a^	9.48 ± 0.52^a^	94.28 ± 0.15^a^	47.40 ± 0.15^a^
Positive control	31.30 ± 0.67^a^	68.50 ± 0.75^d^	16.90 ± 0.86^c^	116.70 ± 0.92^c^	84.50 ± 0.86^c^
Celery (10%)	33.30 ± 0.82^a^	54.60 ± 0.61^c^	14.10 ± 0.76^b^	102.00 ± 0.85^b^	70.50 ± 0.62^b^
Chicory (10%)	35.30 ± 0.92^ab^	48.60 ± 0.61^b^	15.10 ± 0.15^bc^	99.00 ± 0.85^b^	75.50 ± 0.62^b^
Barley (10%)	39.10 ± 0.51^b^	45.20 ± 0.75^b^	14.92 ± 0.35^b^	99.22 ± 0.95^b^	74.60 ± 0.33^b^
Mixture of all (15%)	40.20 ± 0.79^b^	41.60 ± 0.88^a^	14.76 ± 0.52^b^	96.56 ± 0.57^ab^	73.80 ± 0.16^b^

HDL-c: High-density lipoprotein cholesterol, LDL-c: Low-density lipoprotein cholesterol, VHDL-c: Very low-density lipoprotein cholesterol, Values are mean ± SD. Values in the same column sharing the same superscript letters are not statistically significantly different

## Histopathological examination

**Figure 1:**
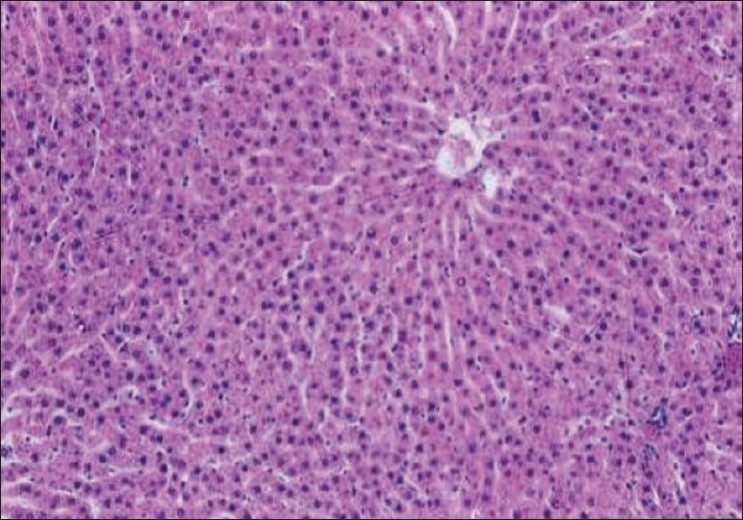
Liver of control C-ve (normal) rats showing normal histology of hepatic lobule. (H and E, ×100)

**Figure 2:**
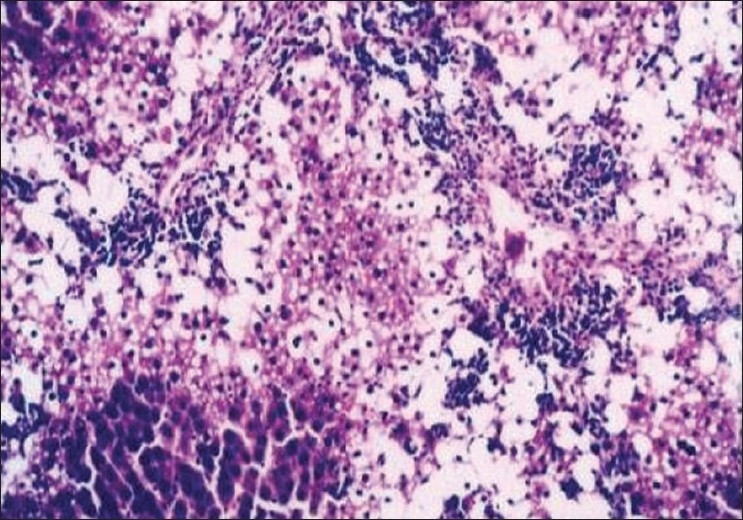
Liver of hypercholesterolemic (control C+ ve) rats showing severe fatty degeneration of hepatocytes and infiltration of leucocytes in hepatic sinusoid. (H and E, ×100)

**Figure 3:**
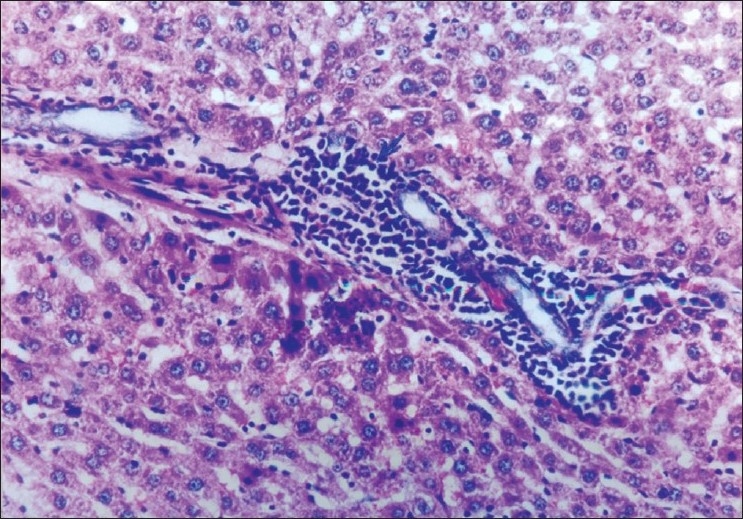
Liver of rats fed on basal diet containing 10% celery powder showing little vacuolar degeneration of hepatocytes mild fatty degeneration of hepatocytes. (H and E, ×100)

**Figure 4:**
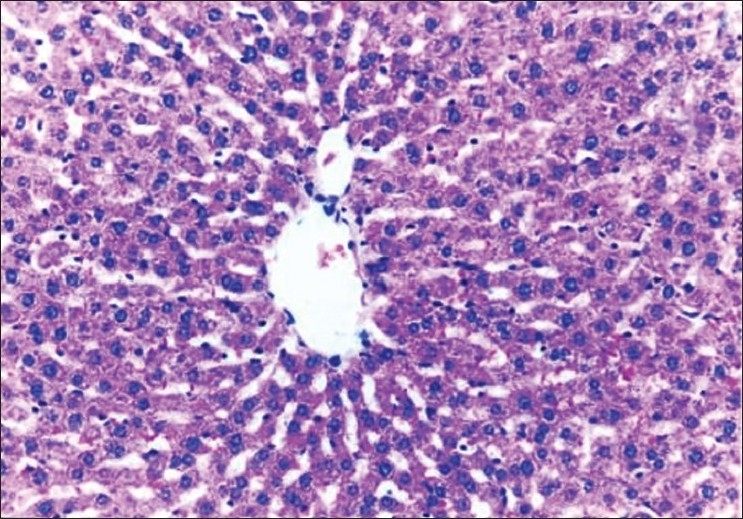
Liver of rats fed on basal diet containing 10% chicory powder showing only mild fatty degeneration of hepatocytes (H and E, ×100)

**Figure 5:**
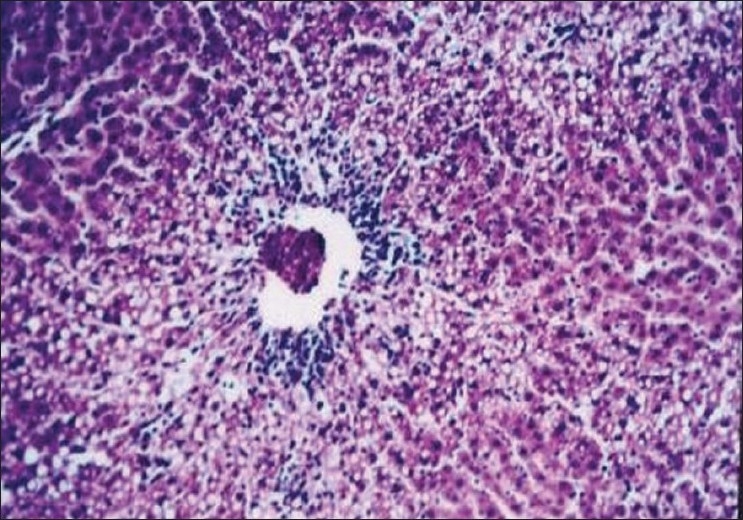
Liver of rats fed on basal diet containing 10% barley powder showing little vacuolar degeneration of hepatocytes and mild leukocytic infiltration around central vein (H and E, ×100)

**Figure 6:**
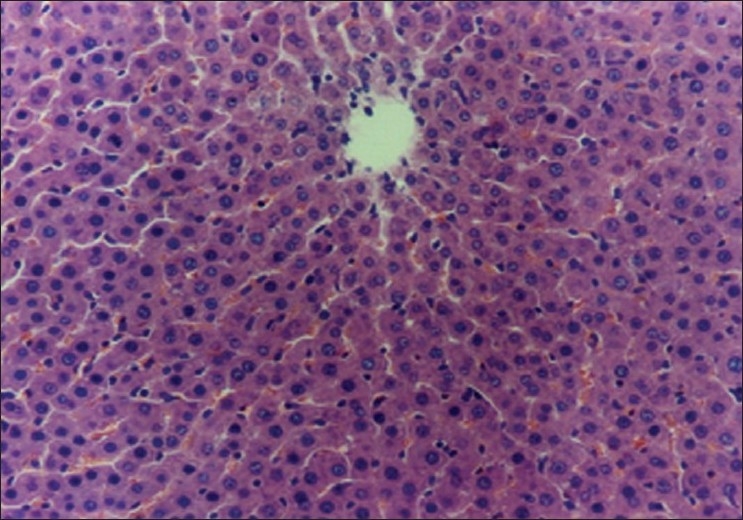
Liver of rats fed on basal diet containing 15% mixture of the three plant materials showing almost normal histology of hepatic lobule. (H and E, ×100)

The biochemical observations reported in this study were supplemented by histopathological examination of liver sections of hypercholesterolemic rats. The obtained results showed that examination of livers of the normal (negative control -ve) rats fed on the basal diet had normal histological picture of hepatic lobule that consists of central vein surrounded by normal hepatocytes [Figure 1]. Examination of liver of hypercholesterolemic rats showed severe fatty degeneration of the hepatocytes and infiltration of leucocytes in hepatic sinusoid [Figure 2]. Livers of hypercholesterolemic rats fed on diet containing celery 10% showed little vacuolar degeneration of hepatocytes and mild fatty degeneration of hepatocytes, as shown in Figure 3. Examination of livers of hypercholesterolemic rats fed on diet supplemented with chicory 10% showed only mild fatty degeneration of the hepatocytes, as illustrated in Figure 4. Hypercholesterolemic rats fed on experimental diet containing barley 10% showed little vacuolar degeneration of hepatocytes and mild leukocytic infiltration around central vein [Figure 5]. Examination of livers obtained from hypercholesterolemic rats fed on diet supplemented with a mixture of celery, chicory, and barley at 15% revealed almost normal hepatic lobules, as illustrated in Figure 6.

## DISCUSSION

The finding results of Table 2 was similar to that reported by Urias-Silvas *et al*. who concluded that inulin (fructans) extracted from chicory regulate appetite and has a promising effect on the body weight.[6]

From Table 3, finding that effect of dietary fiber supplementation was similar to that reported by Tsi and Tan, for celery extracts and by increasing bile acid secretion.[2,19,20]

For chicory leaves and barley grains, the previous studies reported that different extracts of celery leaves or chicory leaves or barley grains effectively lower the elevated serum levels of AST, ALT, and ALP enzymes.[2,19–21]

From Table 4, finding that the cholesterol lowering effect of celery reported in this study was similar to that previously reported by Tsi and Tan. Moreover, the same concluded that the mechanism underlying the hypocholesterolemic activity of celery extracts (aqueous and butanol) could be possibly due to presence of sugar or amino acid side chains(s) compounds. Also, celery contains vitamin C which is a known immune system booster and reduces the free radicals in the body. It also reduces the risk of asthma, osteoarthritis, and rheumatoid arthritis. To top it off, the vitamin is also thought to increase heart health.[19]

The results of the present work about the effectiveness of celery agreed with Kim and Shin, who demonstrated that the lipid-lowering action of this natural product may be mediated through inhibition of hepatic cholesterol biosynthesis, increased faucal bile acids excretion, and enhanced plasma lecithin: cholesterol acyltransferase activity, and reduction of lipid absorption in the intestine.[22]

The potassium and sodium in celery juice help to regulate body fluid and stimulate urine production, making it an important help to rid the body of excess fluid.[23]

The rich presence of tannins in chicory can keep the level of LDL (harmful) cholesterol in check. This can keep our heart healthy.[24]

In addition, the chicory has been used in the folk medicine of Pakistan for treating liver disease and hepatic system-related disorders. A group of clinical researchers recently isolated a phenolic compound called esculetin from the extracts of the chicory, and they further confirmed that it is a hepatoprotective compound. The compound showed liver-protecting activity in mice with hepatic damage induced by paracetamol and carbon tetrachloride. This extract inhibited the oxidative degradation of DNA in tissue debris of mice liver. In addition, in high content of soluble fiber, inulin, chicory can reduce strain on liver by removing extra water and toxin.[25] Inulin is soluble in water and not hydrolyzed by human digestive enzymes; it is expected to behave like a soluble fiber and to have a hypolipidemic effect.[26]

This might treat jaundice, hepatitis, hepatic congestion, etc. Also by stimulating the flow of bile, chicory is considered to treat gallstones, biliary insufficiency, gastritis, and splenomegaly.[2,3]

Inulin lowers serum cholesterol when added to the diet of rats, may decrease cholesterol synthesis by inhibiting hydroxymethylglutaryl-CoA reductase. A mechanism of action of oligofructose was associated with the modulation of *de novo* cholesterol synthesis by short-chain fatty acids produced by the gut microflora during the fermentation process.[27]

Concerning barley, barley may be used as a part of the vegetarian diet because it decreases serum total lipids. Moreover, whole-grain barley is lower in fat, protein, and calories, and higher in total dietary fiber that increasing whole-grain consumption can reduce the risk of coronary heart disease, and can help with weight maintenance.[8]

Barley has a concentration of soluble fibers called β-glucan. These effects are associated with increased excretion of bile acids and neutral sterols, increased catabolism of cholesterol, and reduced absorption of cholesterol and fat.[28]

This finding is in agreement, to some extent, for celery and for chicory in rats. Chicory is purported to have several health benefits. The short-chain fatty acids produced through the fermentation of soluble fiber in the large intestine serve to stabilize blood glucose levels, lower LDL or “bad” cholesterol in the blood, increase the production of immune cells, the concentrated barley beta-glucan in hypercholesterolemic men and women.[29,30]

Histopathological examination of liver sections of hypercholesterolemic rats was nearly similar to histopathological findings obtained in CCl4-hepatotoxic rats when given orally chicory extracts. No available literatures on the effects of celery and barley on the histology of liver could be obtained.[31]

## CONCLUSION

Dietary intake of plant mixture of celery, chicory, and barley at 15% for four weeks may be beneficial for patients suffering from hypercholesterolemia and liver disease as it lowers the elevated serum liver enzymes, total cholesterol, and triglycerides, and improves lipid profile in cholesterol-fed rats. Moreover, diet supplementation with this plant mixture produces an excellent effect on the histology of liver as it ameliorates the hepatic damage seen in the liver of hypercholesterolemic rats.
